# Molecular orbital analysis of the hydrogen bonded water dimer

**DOI:** 10.1038/srep22099

**Published:** 2016-02-24

**Authors:** Bo Wang, Wanrun Jiang, Xin Dai, Yang Gao, Zhigang Wang, Rui-Qin Zhang

**Affiliations:** 1 Institute of Atomic and Molecular Physics, Jilin University, Changchun 130012, China; 2 Jilin Provincial Key Laboratory of Applied Atomic and Molecular Spectroscopy (Jilin University), Changchun 130012, China; 3 Institute of Theoretical Chemistry, Jilin University, Changchun 130023, China; 4 Department of Physics and Materials Science and Centre for Functional Photonics (CFP), City University of Hong Kong, Hong Kong SAR, China

## Abstract

As an essential interaction in nature, hydrogen bonding plays a crucial role in many material formations and biological processes, requiring deeper understanding. Here, using density functional theory and post-Hartree-Fock methods, we reveal two hydrogen bonding molecular orbitals crossing the hydrogen-bond’s O and H atoms in the water dimer. Energy decomposition analysis also shows a non-negligible contribution of the induction term. Our finding sheds light on the essential understanding of hydrogen bonding in ice, liquid water, functional materials and biological systems.

Hydrogen bonding (H-bonding) is an essential interaction in nature and plays a crucial role in physical, chemical and biological processes. One can mention numerous examples such as the role of H-bonding in water^1^, proton transfer processes^2^,^3^, enzymatic catalysis^4^,^5^, protein folding^6^, and also its important role in other fields. Consequently, the ability to understand H-bonding is of great relevance to a variety of problems in science. The water dimer (H_2_O)_2_ is one of the most typical models for studying the H-bonding system and, as such, much scientific effort has been directed toward understanding its properties^7^,^8^,^9^. Several studies have indicated that H-bonds have covalent-like characteristics^10^,^11^,^12^. Recent experiments have not only revealed that the covalent-like characteristics of H-bonds exist between two 8-hydroxyquiline molecules assembled on a Cu(111) substrate^13^, but also directly visualized the frontier molecular orbitals (MOs) of the adsorbed water^14^. In addition, a recent proton nuclear magnetic resonance experiment had also confirmed the covalency of H-bonds in liquid water^15^. Further, our previous theoretical calculations have shown that the delocalized MOs exist in water rings^16^,^17^. These studies help to understand H-bonding from the perspective of MOs.

The (H_2_O)_2_ is the simplest water cluster and the spatial conformation benchmark for studying complex H-bonding systems. Its H-bonding conformation has been frequently studied both experimentally and theoretically^8^,^9^,^18^,^19^,^20^. The H-bond is nearly linear in the (H_2_O)_2_ and its quantum tunneling^21^ and spectroscopy^22^,^23^,^24^ have been studied. Among the notable examples, the OH-stretching vibrations have been studied by matrix-isolation spectroscopy in infrared spectrum of (H_2_O)_2_^25^. Further, OH-stretching bonds have been reproduced theoretically^26^. Moreover, the overtone spectrum can provide a good prediction to the experimental studies^27^; this spectrum has also been observed in atmosphere^28^. There is no direct investigation on MO of the H-bonded (H_2_O)_2_. In contrast, detailed works have been devoted to interaction strength of (H_2_O)_2_, including its the H-bond strength^29^, interaction energy^9^, dissociation energy^30^. However, more comprehensive studies are still needed since the fundamental mechanism of interaction between two water molecules is still not clearly understood. Inspired by a recent study on covalent-like characteristics in the H-bonds between two 8-hydroxyquinoline molecules revealed in an experiment using atomic force microscopy, which were identified to originate from both the covalent charge in H···N and the charge transferred from H to N and O^13^, much experimental and theoretical researches could be done to further explore the intermolecular interaction mechanism of (H_2_O)_2_.

In this work, we present a study aiming to understand the H-bonding mechanism of (H_2_O)_2_ from the MO perspective, which allows illustration of the nature of molecular interaction^11^,^31^,^32^,^33^. For instance, the guanine quartet intermolecular interaction can be presented from MO perspective^34^. The halogen-bonded trihalides DX···A and H-bonded complexes DH···A (D, X, A = F, Cl, Br, I) all have obvious MOs interaction^35^. The combination of orbital morphology with orbital composition offers an intuitive visualization and a qualitative interpretation.

## Results

**Figure 1:**
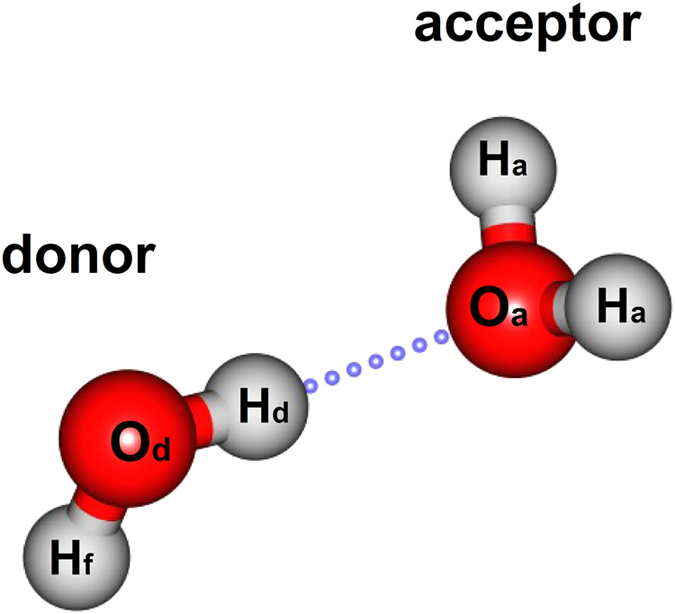
The equilibrium structure of (H_2_O)_2_.

The optimized lowest energy structure of (H_2_O)_2_ is displayed in Fig. 1. For convenience, the geometrical details are given for the following discussion.

**Figure 2:**
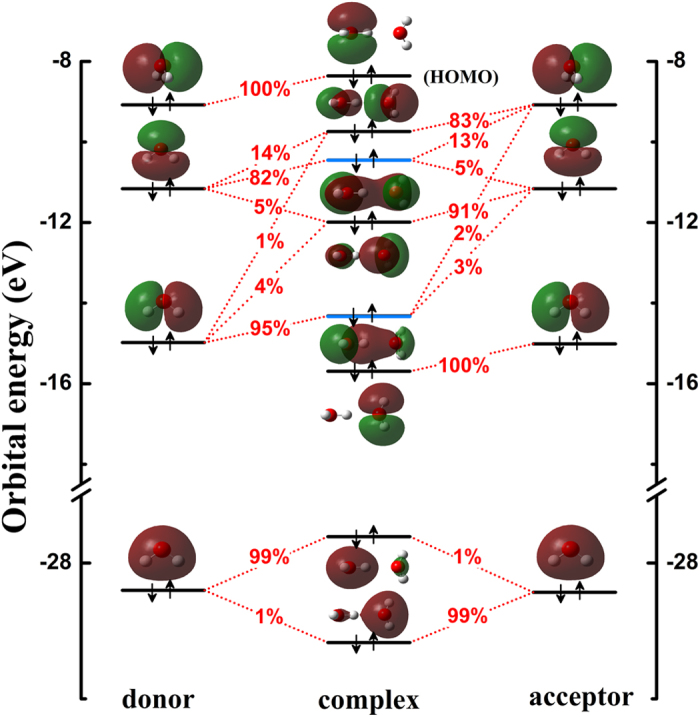
The orbital interaction diagram of (H_2_O)_2_. Orbital energy levels are represented as solid bars. The bars on the left and right sides correspond to the FOs of the two water monomers; the bars in the middle correspond to the complex orbitals of (H_2_O)_2_. The topmost solid black bars denote the highest occupied MOs (HOMOs). Blue solid bars denote two H-bonding MOs between the two water monomers, HOMO-2 and HOMO-4. Two corresponding bars are linked by short red dotted lines in the center of which the component percentage values (%) are given for those with the composition of a FO in a complex orbital larger than 0.5%.

The bonding property of the intermolecular interaction system is effectively revealed by MO analyses^11^,^33^,^34^,^35^, even including the H-bonding interaction between organic molecules^11^,^34^. In this work, we aimed to understand the most typical H-bonding system of (H_2_O)_2_ from the MO perspective. As shown in Fig. 2. The quantitative contributions (in percentages) from atomic orbitals to these complex MOs are also given. Here, the orbital interaction diagram of (H_2_O)_2_ is from calculations at the DFT-PBE0 level^36^,^37^. The PBE0 functional has been shown to give orbital diagrams consistent with results using another ab initio method (see Supplementary Fig. S1). When the contribution from a fragment orbital (FO, i.e. the MO of the water monomer) to a complex orbital is larger than 0.5%, the two energy levels respectively corresponding to FO and the complex orbital are linked in Fig. 2. As is shown, two MOs (HOMO-2 and HOMO-4) clearly cross the region between the two water monomers. The HOMO-2 of (H_2_O)_2_ is formed by mixing FO HOMO-1 (82%) in the donor molecule with FO HOMO-1 (5%) and HOMO (13%) in the acceptor molecule. The HOMO-4 of (H_2_O)_2_ is formed by mixing FO HOMO-2 (95%) in the donor molecule with FO HOMO-1 (3%) and HOMO (2%) in the acceptor molecule. These two crossing MOs are mainly composed of the 2p orbital of O and 1s orbital of H of the donor molecule, with certain contribution from the acceptor molecule (see Fig. 1 for notations of the atoms and Supplementary Table S1 for their contributions in percentages). Moreover, we also analyzed the LUMO of the complex which is about 7.7 eV higher than HOMO, confirming that the electronic structure of the (H_2_O)_2_ is reasonably stable. And the analysis also shows that the LUMO of the complex is mainly from the LUMO of the acceptor water molecule. Similarly, this MOs mixing involved in water has been anticipated in the literature^38^,^39^. Recently, one of us reported a study on the origin of weak interaction in the benzene-methane complex, revealing strong orbital deformation due to CH-π interaction^40^. In the water dimer studied in this work, as a stronger intermolecular interaction system, the direct orbital interaction between two water molecules are also observed as evidenced in the overlap of particular monomeric orbitals. We further obtained a bond order of 0.03 from atom-atom-overlap weighted natural atomic orbital bond order^41^ analysis and 0.08 from Mayer bond order^42^ analysis of the water dimer.

**Figure 3:**
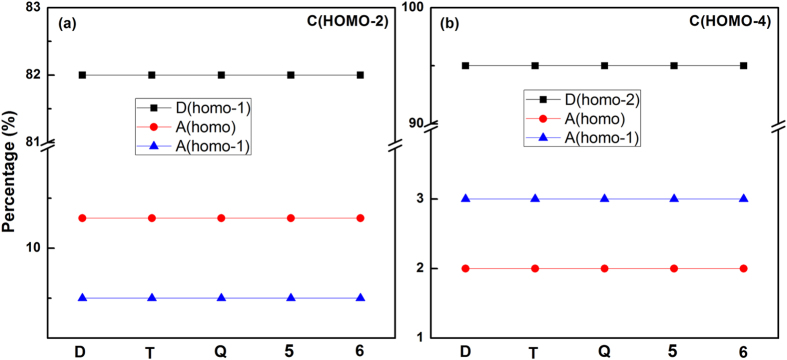
The component percentages of fragment orbitals in the two crossing complex MOs(HOMO-2 (**a**) and HOMO-4 (**b**)), respectively. The Mos of (H_2_O)_2_ are obtained at different levels ofbasis sets through single point calculations at aug-cc-pVXZ (X = D, T, Q, 5 and 6) levels of theory. The symbol C, D and A represent the complex, donor and acceptor waters, respectively. The “homo” denotes the HOMO of water monomer.

Moreover, we analyzed the MOs of (H_2_O)_2_ using different levels of basis sets through single point calculations at aug-cc-pVXZ (X = D, T, Q, 5 and 6) levels of theory. As shown in Fig. 3, the component percentages of fragment orbitals in two crossing complex MOs (HOMO-2 and HOMO-4) are the same. The corresponding isosurfaces of all complex orbitals also show no noticeable variation with the use of different basis sets, as seen in Supplementary Table S2. Meanwhile, we also calculated the electrostatic potential of (H_2_O)_2_ (see Supplementary Fig. S2). The result shows that the charge distribution trends are consistent with previous reports^43^,^44^.

To achieve further insight into the interaction between water monomers, we additionally analyzed the composition of the H-bond of (H_2_O)_2_ using the SAPT treatment. The total interaction energy (E_int_) between two water molecules can be decomposed as:


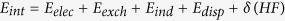


where E_elec_ describes the classical Coulomb interaction between water monomers; E_exch_ is the exchange-repulsion term; E_ind_ is the energy of interaction of the permanent multipole moments of one monomer and the induced multipole moments of the other. This term is interpreted as orbital interaction^11^,^45^, representing the polarization of the electron density between water monomers; E_disp_ is the dispersion interaction energy; the δ(HF) term is a Hartree-Fock (HF) correction for higher-order contributions to the total interaction energy obtained at HF level (further details are listed in Part 5 of the Supplemental Information).

We adopted the PBE0 exchange-correlation functional with the aug-cc-pVQZ basis set for energy decomposition. We used the ionization potential of 0.4638 a.u.^46^ in calculations with asymptotic correction. The SAPT (HF) and SAPT (DFT) interaction energy decompositions for the (H_2_O)_2_ are shown in Table 1. The consistency of the two data sets confirms the unique trend we demonstrate. It can be seen that the E_elec_ term is the most important contribution to E_int_, about twice as large as the total interaction energy and greater than 60% of attractive interaction energy. The E_disp_ term makes certain contributions to total interaction energy. Importantly, the E_ind_ term plays a non-negligible role in stabilizing the (H_2_O)_2_, which amounts to more than 10% of the attractive interaction energy and about 20% of E_disp_.

**Figure 4:**
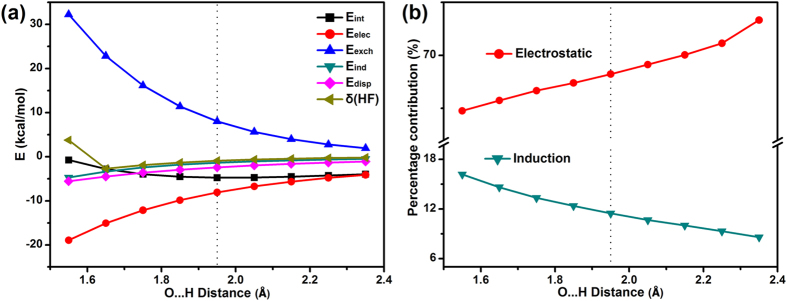
Energy and percentage values of (H_2_O)_2_ at different O…H distances (D_O_…H). (**a**) Interaction, electrostatic, exchange, induction, and dispersion energies of (H_2_O)_2_ at different O…H distances (D_O_…_H_). (**b**) The percentage values represent contribution to the total attractive interactions. The black dotted lines represent the equilibrium O…H distance of (H_2_O)_2_.

The above energy decomposition analysis shows that the system has some orbital overlap. E_ind_ should be intermolecular distance dependent. The greater the distance between molecules, the less orbital overlap there should be, leading to a reduced E_ind_. Therefore, we further analyzed the energy decomposition results at different O…H distances in order to confirm the reliability of our results. The energy decomposition result is given in Fig. 4. Fig. 4(a) presents the interaction, electrostatic, exchange, induction, and dispersion energies of (H_2_O)_2_ at different O…H distances (D_O_…_H_), while Fig. 4(b) shows the corresponding percentage contribution to the total attractive interactions. The results show that with the increase of the O…H distances, the contribution from the electrostatic interaction term gradually increases, and the contribution from the induction term decreases accordingly. The trend is particularly clear in Fig. 4(b), where the change in the percentage contribution of the interaction energy shows that the induction decreases monotonically as the distance increases, reflecting the weakening of the bonding. This trend is reasonable and consistent with our previous report on the water tetramer^17^ and other work^47^. The results further prove that this work may have a fundamental significance in understanding water clusters and complex H-bonding systems.

To further confirm the orbital interaction of H-bonding in (H_2_O)_2_, we calculated proton shielding tensor for (H_2_O)_2_. The results are shown in Table S3 of Supplementary Material. The perpendicular component σ^⊥^ of proton magnetic shielding tensor has characterized the orbital interaction of H-bond^15^. In addition, the previous studies shows that the σ^⊥^ of proton magnetic shielding tensor is 17.9 ppm for liquid water at 80 °C, which revealed the orbital interaction of H-bond^15^,^48^. It is seen that our σ^⊥^ of proton magnetic shielding tensor of 17.9 ppm is the same. Thus, this result is in good agreement with experimental observations and theoretical calculations, and further confirms the reliability of our calculation results.

## Discussion

The calculated electronic structure illustrates the orbital interaction between water monomers in (H_2_O)_2_. There are two MOs crossing the (H_2_O)_2_ system along the H-bonding region. Furthermore, the energy decomposition analysis demonstrates that E_ind_ is non-negligible in the interaction between two monomers. Recently, the electronic structure of the H-bond has been visualized by low-temperature scanning tunneling microscope^14^. This study provides a critical insight at the atomic level for understanding H-bonding systems and the prediction of their properties.

## Methods

To achieve insightful MO analysis of the interaction systems, we adopted density functional theory (DFT) to obtain accurate geometric parameters and further visualization of Kohn-Sham MOs^11^,^49^. We used the coupled cluster singles, doubles, and perturbative triples [CCSD(T)] levels of theory^50^,^51^,^52^ to validate our DFT results. The latter is known to provide results in excellent agreement with experimental data^22^. For the DFT calculations, we chose to use a PBE0 functional as it has been widely used in describing H-bonding interactions and is capable of offering a good performance for treating H-bonds^53^. As such, we have optimized the (H_2_O)_2_ at both CCSD(T) and PBE0 levels with anaug-cc-pVQZ basis set^54^ using Gaussian 09^55^. We further performed energy decomposition based on symmetry-adapted perturbation theory (SAPT) using the Molpro 2012 program^56^. The basis set superposition error was calculated using the counterpoise method. Considering the good agreement between the SAPT (CCSD) and SAPT (DFT) results, which give quite similar energy components in nearly all cases^57^, we performed SAPT(DFT) calculations of (H_2_O)_2_ using δ(HF) correction.

## Additional Information

**How to cite this article**: Wang, B. *et al.* Molecular orbital analysis of the hydrogen bonded water dimer. *Sci. Rep.* **6**, 22099; doi: 10.1038/srep22099 (2016).

## Supplementary Material

Supplementary Information

## Figures and Tables

**Table 1:** SAPT interaction energy (kcal/mol) decomposition results for (H_2_O)_2_.

	SAPT(HF)	SAPT(DFT)
E_elec_	−8.37	−8.10
E_exch_	7.04	8.04
E_ind_	−1.35	−1.37
E_disp_	—	−2.41
δ(HF)	−0.92	−0.92
E_int_	−3.60	−4.76(−4.95^a^)

^a^denotes the interaction energy calculated at CCSD(T)/aug-cc-pVQZ level.
