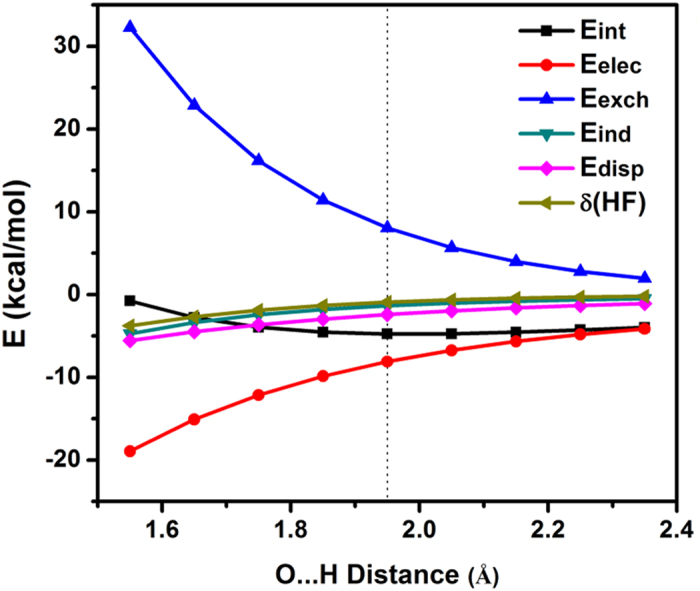# Corrigendum: Molecular orbital analysis of the hydrogen bonded water dimer

**DOI:** 10.1038/srep29148

**Published:** 2016-07-08

**Authors:** Bo Wang, Wanrun Jiang, Xin Dai, Yang Gao, Zhigang Wang, Rui-Qin Zhang

Scientific Reports 6: Artcile number: 22099; 10.1038/srep22099Published online: 02242016; Updated: 07082016

In this Article, there is an error in Figure 4a. The sign of “E (kcal/mol)” was inverted for the leftmost data point for δ(HF); −4 (approximately) was incorrectly plotted as +4 (approximately). The correct Figure 4 appears below as Figure 1. The Figure legend is correct.

## Figures and Tables

**Figure 1:**