# Supplementary material for: Molecular orbital analysis of the hydrogen bonded water dimer
Source: Sci Rep. 2016 Feb 24;6:22099. doi: 10.1038/srep22099 (PMC4764947; doi:10.1038/srep22099)
Supplement: Supplementary Information [file srep22099-s1.pdf]

Supplementary Information

Molecular orbital analysis of the hydrogen bonded water dimer

Bo Wang,[1,2] Wanrun Jiang,[1,2] Xing Dai,[1,2] Yang Gao,[1,2] Zhigang Wang*[1,2,3] and Rui-Qin Zhang*[4]

[1] Bo Wang, Wanrun Jiang, Xing Dai, Yang Gao, Zhigang Wang
Institute of Atomic and Molecular Physics, Jilin University, Changchun 130012, China. E-mail: wangzg@jlu.edu.cn
[2] Bo Wang, Wanrun Jiang, Xing Dai, Yang Gao, Zhigang Wang
Jilin Provincial Key Laboratory of Applied Atomic and Molecular Spectroscopy (Jilin University), Changchun 130012, China
[3] Zhigang Wang
Institute of Theoretical Chemistry, Jilin University, Changchun 130023, China
[4] Rui-Qin Zhang
Department of Physics and Materials Science and Centre for Functional Photonics (CFP), City University of Hong Kong, Hong Kong SAR, China. E-mail: aprqz@cityu.edu.hk

Contents

Part 1. The orbital interaction diagram of $(H_2O)_2$ at HF level based on CCSD(T) calculations.

Part 2. The contribution percentages from atomic orbitals to complex MOs.

Part 3. The isosurfaces of all complex orbitals at the PBE0/aug-cc-pVXZ levels (X=D, T, Q, 5 and 6).

Part 4. The electrostatic potential distributed on the vdW surface of $(H_2O)_2$.

Part 5. More details of interaction energy decomposition.

Part 6. The proton magnetic shielding tensor of $(H_2O)_2$.

Part 1. The orbital interaction diagram of $(H_2O)_2$ at HF level based on CCSD(T) calculations.

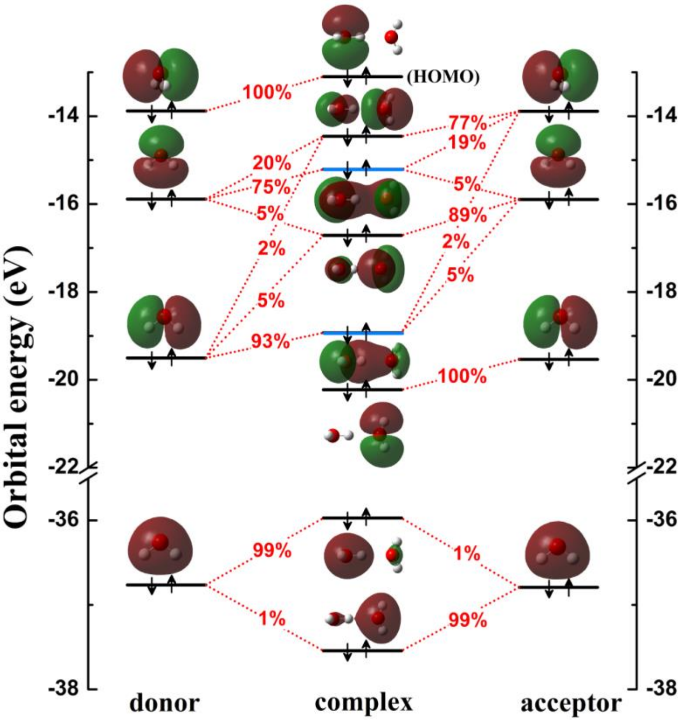

Supplementary Figure S1 The orbital interaction diagram of $(H_2O)_2$. Orbital energy levels are represented as solid bars. The bars on the left and right columns correspond to the fragment orbitals (FOs) of the two water monomers; the bars in the middle column corresponds to complex orbitals of $(H_2O)_2$. The topmost black solid bars denote the highest occupied MOs (HOMOs). Blue solid bars denote two H-bonding MOs between two water monomers, HOMO-2 and HOMO-4. Two corresponding bars are linked by red short dot lines and the component percentage values (%) are given for those with the composition of a FO in a complex orbital larger than 0.5%.

To clarify that the orbital diagram presented in the manuscript is consistent with results using other ab initio methods, we calculated the orbital interaction of $(H_2O)_2$ at HF level based on CCSD(T) calculations. When the contribution component of a fragment orbital (FO, i.e. the MO of water monomer) to a complex orbital is larger than 0.5%, the two energy levels respectively corresponding to FO and complex orbital are linked in Supplementary Figure S1. Here we found that there are two MOs (HOMO-2 and HOMO-4) clearly cross the region between the two water monomers. The HOMO-2 of $(H_2O)_2$ is formed by mixing FO HOMO-1 (75%) in the donor molecule with FO HOMO-1 (5%) and HOMO (19%) in the acceptor molecule. The HOMO-4 of $(H_2O)_2$ is formed by mixing FO HOMO-2 (93%) in the donor molecule and the FO HOMO-1 (5%) and HOMO (2%) in acceptor molecule.

Part 2. The contribution percentages from atomic orbitals to complex MOs.

Supplementary Table S1. MO components of $(H_2O)_2$. The corresponding atomic labels are given in Figure 1. Results in bold denote the two crossing MOs.

$(H_2O)_2$ (%)	O^d		H^f	H^d	O^a		H^a	H^a
	2s	2p	1s	1s	2s	2p	1s	1s
HOMO	0.00	99.38	0.00	0.00	0.00	0.00	0.01	0.01
HOMO-1	0.70	14.71	0.37	0.05	0.01	83.60	0.00	0.00
**HOMO-2**	**8.38**	**67.21**	**3.06**	**2.56**	**0.65**	**17.39**	**0.16**	**0.16**
HOMO-3	0.91	6.24	1.09	0.02	8.89	76.26	3.08	3.08
**HOMO-4**	**0.00**	**69.04**	**12.74**	**12.77**	**0.11**	**4.64**	**0.16**	**0.16**
HOMO-5	0.00	0.01	0.00	0.00	0.00	73.59	13.01	13.01
HOMO-6	75.79	3.16	9.44	9.75	0.42	0.11	0.07	0.07
HOMO-7	0.35	0.07	0.03	0.28	76.32	3.08	9.35	9.35

Part 3. The isosurfaces of all complex orbitals at the PBE0/aug-cc-pVXZ levels (X=D, T, Q, 5 and 6).

Supplementary Table S2. The isosurfaces of all complex orbitals at the PBE0/aug-cc-pVXZ levels (X=D, T, Q, 5 and 6).

aug-cc-pVDZ	aug-cc-pVTZ	aug-cc-pVQZ	aug-cc-pV5Z	aug-cc-pV6Z

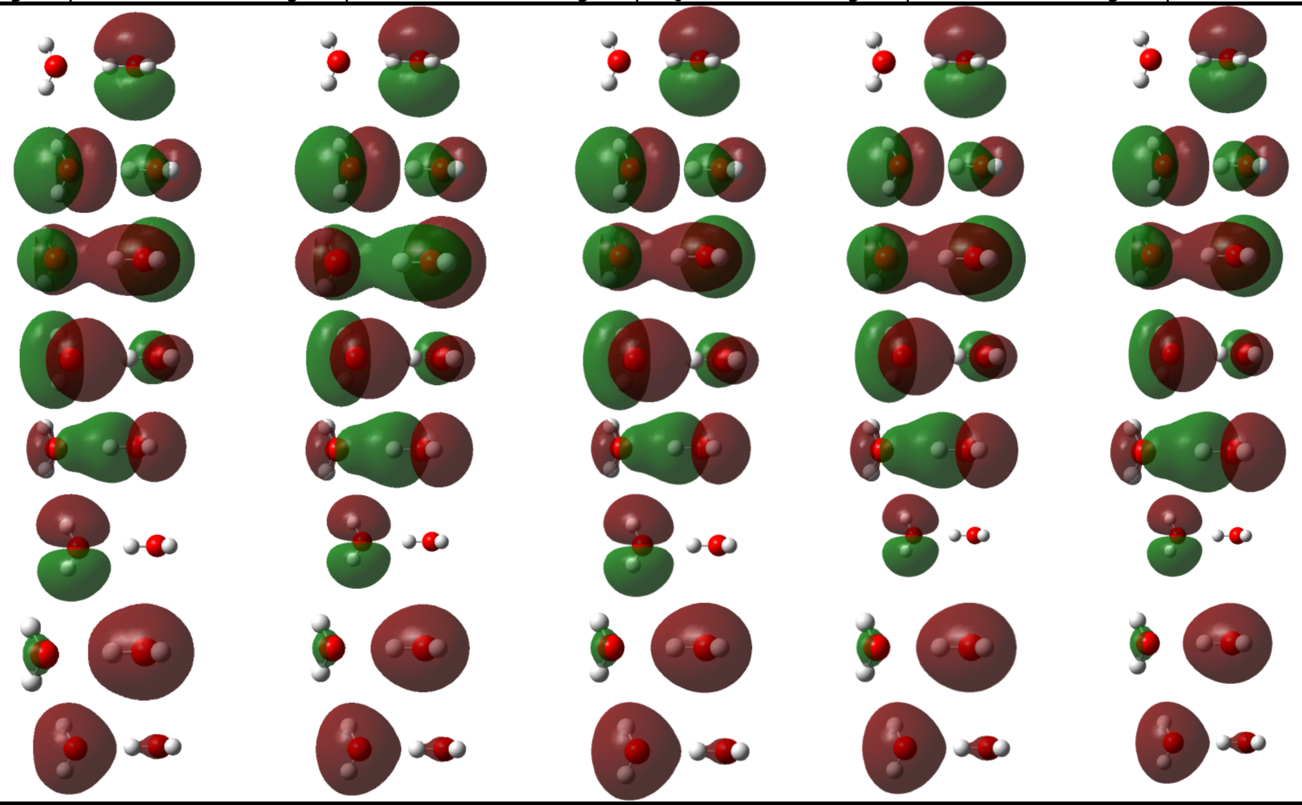

Part 4. The electrostatic potential distributed on the vdW surface of $(H_2O)_2$.

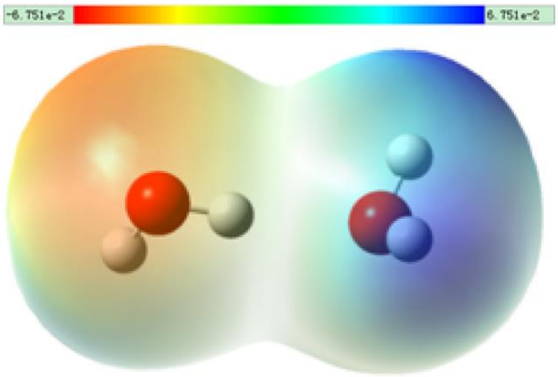

Supplementary Figure S2. The electrostatic potential distributed on the vdW surface of $(H_2O)_2$

Part 5. More details of interaction energy decomposition.

In the SAPT method, the total interaction energy, E_{int} is given as the sum of first-order energy (E^1) and second-order (E^2) and $\delta(\mathrm{HF})$ term, E^1_{pol} is electrostatic interaction term, E^1_{exch} is exchange-repulsion term, E^2_{ind} is induction term. $E^2_{ind-exch}$ is exchange-induction term, E^2_{disp} is dispersion term, $E^2_{disp-exch}$ is exchange-dispersion term. The $\delta(\mathrm{HF})$ term is a Hartree–Fock correction for higher-order contributions to the interaction energy. These interaction energy components can be calculated according to the equations (1):

$$E_{elec} = E^1_{\mathrm{pol}}$$

$$E_{exch} = E^1_{exch}$$

$$E_{ind} = E^2_{ind} + E^2_{ind-exch} \quad (1)$$

$$E_{disp} = E^2_{disp} + E^2_{disp-exch}$$

$$E_{int} = E_{elec} + E_{exch} + E_{ind} + E_{disp} + \delta(\mathrm{HF})$$

Part 6. The proton magnetic shielding tensor of $(H_2O)_2$.

Supplementary Table S3. Calculated proton magnetic shielding tensor (ppm).

Gometry	σ_{iso}	$\Delta\sigma$	$\sigma_{\perp}$
$(H_2O)_2$	27.9	20.1	17.9
Liquid at 80 ℃[a]	26.2	25.1	17.9
Liquid at 27 ℃[a]	25.7	27.4	16.1
Liquid at 0 ℃[a]	25.4	28.5	15.9
Ice Ih[a]	21.1	34.2	9.7

[a]presents the ref. 16 and 43
